# Synthesis of Homoveratric Acid-Imprinted Polymers and Their Evaluation as Selective Separation Materials

**DOI:** 10.3390/molecules16053826

**Published:** 2011-05-05

**Authors:** Mariusz Dana, Piotr Luliński, Dorota Maciejewska

**Affiliations:** Department of Organic Chemistry, Faculty of Pharmacy, Medical University of Warsaw, Banacha 1, 02-097 Warsaw, Poland; Email: mariusz.dana@wum.edu.pl (M.D.); piotr.lulinski@wum.edu.pl (P.L.)

**Keywords:** molecularly imprinted polymers, homoveratric acid, molecular modeling, molecular recognition

## Abstract

A bulk polymerization method was used to easily and efficiently prepare homo-veratric acid (3,4-dimethoxyphenylacetic acid)-imprinted polymers from eight basic monomers: 2-vinylpyridine, 4-vinylpyridine, 1-vinylimidazole, *N*-allylaniline, *N*-allyl-piperazine, allylurea, allylthiourea, and allylamine, in the presence of homoveratric acid as a template in *N*,*N*-dimethylformamide as a porogen. The imprinted polymer prepared from allylamine had the highest affinity to the template, showing an imprinting factor of 3.43, and allylamine polymers **MIP8**/**NIP8** were selected for further studies. Their binding properties were analyzed using the Scatchard method. The results showed that the imprinted polymers have two classes of heterogeneous binding sites characterized by two pairs of *K*_d_, *B*_max_ values: *K*_d_(1) = 0.060 μmol/mL, *B*_max_(1) = 0.093 μmol/mg for the higher affinity binding sites, and *K*_d_(2) = 0.455 μmol/mL, *B*_max_(2) = 0.248 μmol/mg for the lower affinity binding sites. Non-imprinted polymer has only one class of binding site, with *K*_d_ = 0.417 μmol/mL and *B*_max_ = 0.184 μmol/mg. A computational analysis of the energies of the prepolymerization complexes was in agreement with the experimental results. It showed that the selective binding interactions arose from cooperative three point interactions between the carboxylic acid and the two methoxy groups in the template and amino groups in the polymer cavities. Those results were confirmed by the recognition studies performed with the set of structurally related compounds. Allylamine polymer **MIP8** had no affinity towards biogenic amines. The obtained imprinted polymer could be used for selective separation of homoveratric acid.

## 1. Introduction

Organic network polymers, capable of recognizing small molecules, are produced by molecular imprinting techniques [1,2]. Molecularly imprinted polymers (MIPs) are synthesized from a functional monomer and a cross-linking agent in presence of a template molecule. The cavities formed in polymer matrix are complementary in both shape and chemical functionality to the target molecule. The bulk polymerization method is used for the easy and efficient preparation of MIPs [3]. Polymeric materials are applied in many areas such as organic synthesis and catalysis [4,5], drug discovery [6], combinatorial chemistry [7] and drug delivery [8]. One of the most important applications of MIPs are chromatographic techniques where they are used as stationary phases, for example in solid phase extraction (SPE) [9,10]. They have been used successfully for clean-up and enrichment of analytic samples containing drugs, their metabolites or biogenic molecules. The high degree of selectivity provided by MIPs confirms their utility for SPE [11,12,13].

Homoveratric acid (3,4-dimethoxyphenylacetic acid) was found to be main urinary metabolite of homoveratrylamine [2-(3,4-dimethoxyphenyl)ethylamine], a potential metabolite of dopamine with two methylated hydroxyl groups in the aromatic ring [14,15]. Homoveratrylamine and homoveratric acid are clinically important compounds, as both have attracted attention as potential neurotoxins involved in schizophrenia [16]. The role of both compounds in the pathogenesis of Parkinson’s disease is still being considered [17,18] and possible metabolism pathways investigated [19]. An increase in urinary excretion of homoveratric acid could be involved in the metabolism of endogenic compounds, and can indicate the progress of neurological diseases, or can be a result of transformation of exogenic compounds such as drugs [20]. Homoveratric acid has also been found also in plants [21].

Our group is engaged in searching for new selective materials for use in the separation and preconcentration of neurotransmitters and their metabolites [22,23]. Detailed studies of the homoveratric acid imprinted polymers are needed for further progress in search for new stationary phases which could be applicable to separate homoveratric acid and finally could help to explain some aspects of dopamine-dependent diseases. 

The rational design of MIPs is not an easy task, because of the number of experimental variables involved. In order to understand the mechanism of recognition in the polymer matrix, many groups have analyzed theoretically the energy of interactions in the prepolymerization complexes (between the template and the monomers) and used it successfully for discussion of MIPs affinity towards the analytes [24,25,26].

The aim of this paper was the synthesis of some homoveratric acid-imprinted polymers and their evaluation as selective separation materials. The theoretical analysis of intermolecular interactions in the corresponding prepolymerization complexes was utilized to rationalize the effect of the functional monomers on the affinity of the resulting polymers towards the template molecule. The non-covalent approach was applied to obtain the bulk polymers from different functional monomers. The polymer with the highest imprinting factor was selected to further analysis. Next, the effects of cross-linkers on the recognition properties were analyzed. Scatchard analysis was employed to examine the binding properties. The morphology of the polymer’s’ surface was analyzed by scanning electron microscopy. Two sets of the non-competitive binding experiments were performed to estimate the selectivity of polymer toward structurally related and biologically active compounds. Finally, solid phase extraction was performed to estimate the ability of the imprinted material to separate homoveratric acid. 

## 2. Results and Discussion

### 2.1. Affinity of Imprinted Polymers Towards Homoveratric Acid

#### 2.1.1. Imprinting Factors

The polymers were prepared from eight functional monomers: 2-vinylpyridine (**1**), 4-vinylpyridine (**2**), 1-vinylimidazole (**3**), *N*-allylaniline (**4**), *N*-allylpiperazine (**5**), allylurea (**6**), allylthiourea (**7**), and allylamine (**8**) in the presence of homoveratric acid acting as the template. The polymerizations were carried out in *N*,*N*-dimethylformamide (DMF) as the porogen and in the presence of ethylene glycol dimethacrylate (EGDMA) as the cross-linker.

**Table 1:** Binding capacities of **MIP1**–**MIP8** and **NIP1**-**NIP8** together with calculated imprinting factors.

**Polymer No.**	Amount of Homoveratric Acid Bound to Polymer ± S.D. [μmol/g]		Imprinting Factor
	MIP	NIP	
**1**	0.61 ± 0.02	0.78 ± 0.02	0.78
**2**	2.23 ± 0.13	2.64 ± 0.12	0.84
**3**	5.03 ± 0.32	5.55 ± 0.46	0.91
**4**	0.49 ± 0.01	0.22 ± 0.01	2.23
**5**	7.25 ± 0.84	8.13 ± 0.89	0.89
**6**	0.98 ± 0.03	0.82 ± 0.03	1.20
**7**	3.84 ± 0.02	3.92 ± 0.18	0.98
**8**	3.46 ± 0.20	1.01 ± 0.06	3.43

Stationary binding experiments (see Experimental) were used for determination of binding properties of the obtained polymer particles **MIP1–MIP8** and **NIP1–NIP8**. The binding capacities (B, μmol/g) of the MIPs and NIPs for a 24 μmol/L standard solution of homoveratric acid were calculated according to Equation (1):

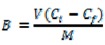
(1)
where *V* represents the volume of standard solution (mL), *C_i_* represents the initial solution concentration (mmol/L), *C_f_* represents the solution concentration after adsorption (mmol/L) and *M* is the mass of polymer particles [27].

The binding capacities of **MIP1–MIP8** were compared to those of **NIP1–NIP8** by calculation of the imprinting factors (IF) according to Equation (2):


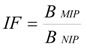
(2)

Both quantities are presented in Table 1. As it can be seen, the four polymers with the highest capacities (**MIP2**, **MIP3**, **MIP5**, and **MIP7**) have no selectivity and their imprinting factors are below 1. The polymer **MIP1** shows both low capacity and lack of selectivity. Only three polymers (**MIP4**, **MIP6**, and **MIP8**) show selectivity towards homoveratric acid and among those, **MIP8** is a most promising one, since it is the only polymer which has both a very high imprinting factor and sufficient binding capacity (see Table 1). In order to rationalize those results on a molecular level, we performed theoretical calculations of the structures and the energies of the prepolymerization complexes. 

#### 2.1.2. Molecular Modeling of Prepolymerization Complexes

Generally, in the prediction of MIP properties it is assumed that the polymer which has the highest affinity towards a given template should have the highest interaction energy between template and the appropriate monomer in the theoretical prepolymerization complex computations [28,29]. This means that the solution prepolymerization structure should be preserved in the polymer matrix. In our discussion we considered two energy parameters: the enthalpies of formation of prepolymeric complexes (Δ*H_complex_*) and the energies of the complexation reaction (Δ*E*) calculated using Equation (3):

Δ*E* = Δ*H_complex_* − Δ*H_homoveratric acid_* − 4Δ*H_monomer_* (3)

**Figure 1:**
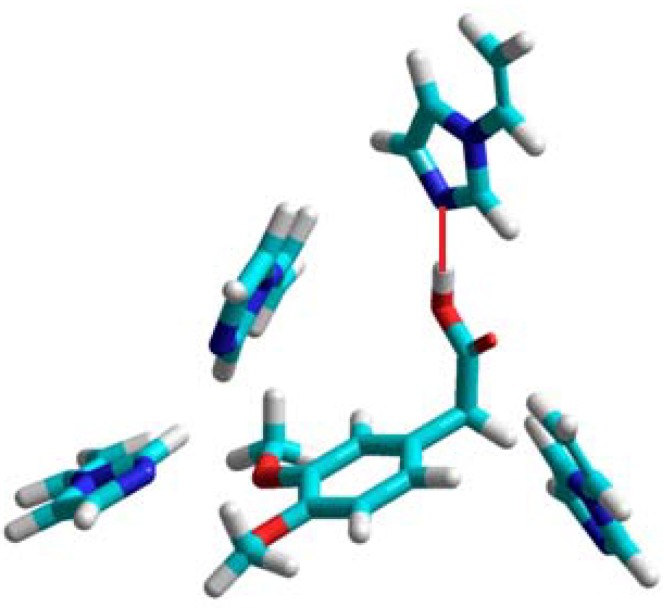
A view of the prepolymerization complex of homoveratric acid with 1-vinyl-imidazole. The hydrogen bond is shown as a red line.

In the computational approach we have analyzed eight functional monomers **1**–**8**. Details of the computational procedures are given in Experimental section. The theoretical computations showed that three monomers: 2-vinylpyridine (**1**), 4-vinylpyridine (**2**), and 1-vinylimidazole (**3**) form unstable complexes with positive enthalpies of formation (Δ*H_complex_*) in the 21 to 43 kcal/mol range. This observation suggests that the monomers **1**–**3** do not provide highly selective binding sites in the resulting polymer matrix, and would not be good candidates to form selective MIPs. Those findings are in good agreement with the experimental studies, which revealed imprinting factors below 1. The weakest complex of homoveratric acid with 1-vinylimidazole is presented in Figure 1. We can observe only one hydrogen bond between the N atom of the imidazole ring and H atom of the carboxylic acid group of homoveratric acid with a length of 1.8 Å. The remaining 1-vinylimidazole molecules do not interact with the template. Weak homoveratric acid complexes with *N*-allylaniline (**4**) and allylthiourea (**7**) are characterized by slightly negative values of the enthalpy of formation (on average −3.5 kcal/mol) and high negative Δ*E* values (on average −22 kcal/mol). The theoretical results seem to indicate that both monomers should form the polymers with low capacity but with imprinting sites. These findings agreed with the analysis of the *N*-allylaniline polymer, but did not agree with the experimental results for allylthiourea polymer. Thorough examination of the prepolymerization complex structure formed by allylthiourea revealed only one intermolecular interaction with the template, and that could be the reason why a lack of the imprinting sites was noted. The simulations for the remaining monomers **5**, **6** and **8** indicate that they form the most stable complexes with negative energies of formation (Δ*H_complex_* equal to −106, −276, −105 kcal/mol, respectively) and negative energy of complexation (Δ*E* equal to −16, −15, −19 kcal/mol, respectively).

*N*-Allylpiperazine (**5**), allylurea (**6**), and allylamine (**8**) should be good candidates for the imprinting procedure. Unfortunately, the experiments did not confirm those findings for the polymer prepared from *N*-allylpiperazine (see Table 1). In the prepolymerization complex formed by homoveratric acid and *N*-allylpiperazine only one NH group of *N*-allylpiperazine is involved in a hydrogen-bonding interaction with the carboxylic group of homoveratric acid, and the lack of selectivity of corresponding polymer towards homoveratric acid indicates that this single interaction is not sufficient to create selective sites in the polymer matrix (like in the case of allylthiourea). The allylurea-based polymer showed some imprinting effect, but the low capacity of this polymer eliminated it from consideration for analytical applications.

**Figure 2:**
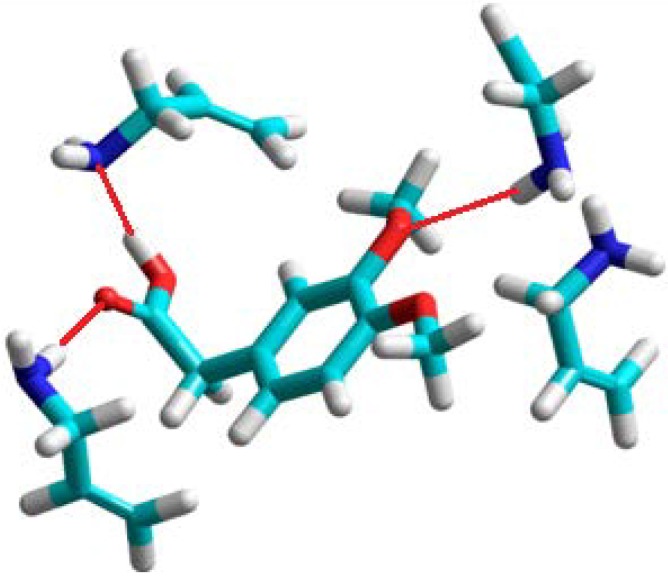
A view of the prepolymerization complex of homoveratric acid with allylamine. Hydrogen bonds are shown as red lines.

The theoretical results showed that allylamine should be the only monomer which forms very stable complexes with many hydrogen-bonding interactions. The structure of the complex is shown in Figure 2. Hydrogen bonds are formed between the carboxylic acid group of template and two allylamine molecules. The remaining two allylamine molecules are in the proximity of the two methoxy groups of homoveratric acid, and one of them forms a hydrogen bond with the O atom. The experiments showed that the allylamine based polymer has the highest imprinting factor, together with high binding capacity.

### 2.2. Evaluation of Imprinting Polymers Using Dynamic Binding Procedure

The dynamic binding method is very similar to the loading step in solid phase extraction and more exactly reflects the ability of imprinted materials to work as a stationary phase. First, we performed the dynamic binding procedure (see Experimental) for the polymers **MIP8** and **NIP8** prepared from allylamine. We carried out the experiments using a 24 μmol/L standard solution of homoveratric acid (the same as in the stationary procedure). The binding capacities (B, μmol/g) of MIP and NIP were calculated according to Equation (4):

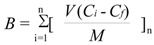
(4)
where *V* represents the volume of portion (mL) in each loading step, n is the number of loading steps performed until no further adsorption on polymer was observed, *C_i_* represents the initial solution concentration (mmol/L), *C_f_* represents the solution concentration after adsorption (mmol/L) and *M* is the mass of polymer particles [23]. 

The results revealed that the binding capacity of **MIP8** increased from 3.46 ± 0.20 to 14.93 ± 0.57 μmol/g, and this fact resulted in a corresponding decrease in selectivity (imprinting factor decreased from 3.43 to 2.45). The results suggest that the binding process involves a non-specific adsorption. 

Next, we employed the dynamic binding procedure to analyze the binding properties of **MIP1–MIP8** and **NIP1–NIP8** at a higher homoveratric acid concentration of 0.35 mmol/L. This concentration was selected because it let us compare data obtained with the experiment performed with the set of structurally related compounds, because some of those compounds demonstrated high LODs and LOQs (see Experimental). The imprinting factors (IF) were calculated according to Equation (2). 

The dynamic binding procedure using a 0.35 mmol/L standard solution of homoveratric acid allowed to increase the binding capacity of all MIPs and NIPs, but produced the disappearance of the imprinting effect (IFs were close to 1) for all polymers except **MIP8**. The binding capacity of **MIP8** was 95.89 ± 3.68 μmol/g and its IF was 1.44. The possible reason for such behavior in the imprinted materials was the increase of non-specific adsorption when the higher concentrations of standard solution were applied on the polymer.

On the basis of those results, the polymer **MIP8** prepared from allylamine, turned out to be the most promising material for separation of homoveratric acid, and it was selected for further optimization of synthetic and analytical procedures.

### 2.3. Effect of Cross-Linker

In order to improve the properties of allylamine polymer, we investigated the effect of the cross-linker used in the polymerization. We synthesized two pairs of MIPs and NIPs from allylamine and two different cross-linkers: **MIP8_a_** (using triethylene glycol dimethacrylate, TGDMA) and **MIP8_b_** (with trimethylolpropane trimethacrylate, TRIM), and then carried out the stationary binding experiments using 24 µmol/L standard solution of homoveratric acid (see Experimental). The results of the analyses are presented in Table 2, together with the data for **MIP8**.

**Table 2:** Binding capacities of **MIP8**, **MIP8_a,b_** and **NIP8**, **NIP8_a,b_** together with calculated imprinting factors.

**Polymer No.**	Amount of Homoveratric Acid Bound to Polymer ± S.D. [μmol/g]		Imprinting Factor
	MIP	NIP	
**8**	3.46 ± 0.20	1.01 ± 0.06	3.43
**8_a_**	1.41 ± 0.05	1.14 ± 0.06	1.24
**8_b_**	4.12 ± 0.57	4.83 ± 0.64	0.85

It was found, that when the the cross-linker TRIM was used to form **MIP8_b_**, the polymeric particles exhibited the highest binding capability, but they did not reveal any imprinting effect. This could be explained by the formation of the more rigid polymer matrix together with a higher specific polymer surface which did not favor specific adsorption. The use of TGDMA as the cross-linker also did not improve the properties of the MIP (see the data for **MIP8_a_**). **MIP8** presents the highest imprinting factor towards homoveratric acid and sufficient binding capacity.

### 2.4. Binding Characteristics

On the basis of previous results, we selected **MIP8** and **NIP8** for a detailed analysis of their binding properties. We employed the Langmuir model transformed to the Scatchard Equation (5):

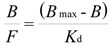
(5)
where *B*_max_ is the total number of the binding sites, *K*_d_ is the dissociation constant, *B* is the bound amount of the analyte, and *F* is the unbound amount of analyte. A system which fits well the Langmuir model gives a straight line in the Scatchard plot, with a slope equal to - (1/*K*_d_) and the *y*-intercept gives *B*_max_/*K*_d_. 

**Figure 3:**
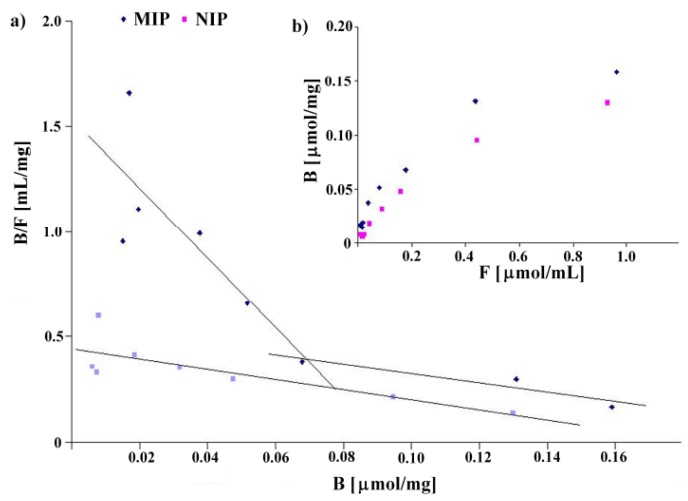
Scatchard plots (**a**) and binding isotherms (**b**) obtained for **MIP8** and **NIP8**.

The binding isotherms were determined by addition of a fixed amount of the polymer to various concentrations of homoveratric acid in the range between 0.019 and 1.2 mmol/L using a stationary binding procedure (see Experimental). The results are shown in Figure 3. The binding isotherms present typical trends for the Langmuir model (see Figure 3b). Linear fits for Scatchard analysis revealed two straight lines for **MIP8** and only one for **NIP8** (see Figure 3a). These results agree with the characteristics of a pair of imprinted and non-imprinted polymers obtained by the non-covalent approach.

**MIP8** has two classes of heterogeneous binding sites characterized by two *K*_d_ and two *B*_max_ values: *K*_d_(1) = 0.060 μmol/mL and *B*_max_(1) = 0.093 μmol/mg for the higher affinity binding sites, and *K*_d_(2) = 0.455 μmol/mL and *B*_max_(2) = 0.248 μmol/mg for the lower affinity binding sites. **NIP8** has only one class of binding site with *K*_d_ = 0.417 μmol/mL and *B*_max_ = 0.184 μmol/mg. Therefore, the experiment clearly showed that the imprinting process had occurred.

### 2.5. Morphology of Particles

**Figure 4:**
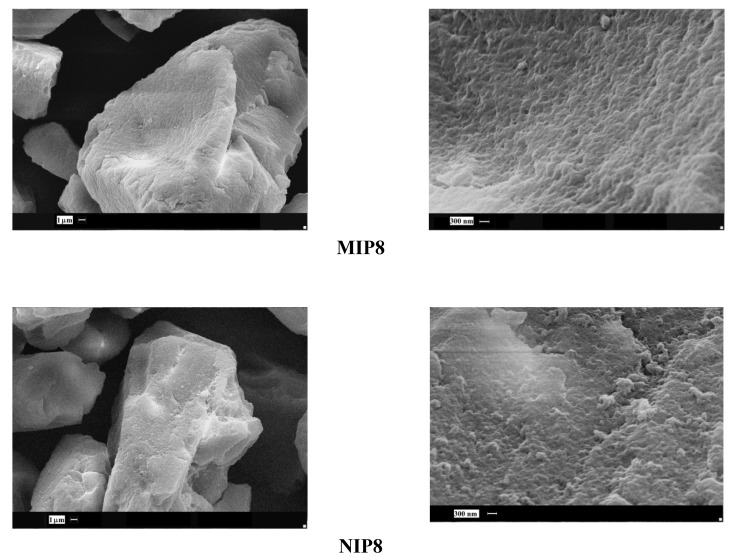
SEM micrographs of **MIP8** and **NIP8** particles.

Scanning electron microscopy was employed to observe the surface of selected particles. First, we observed the surface of a pair of allylamine polymers **MIP8** and **NIP8**. For **MIP8** we observed the highest imprinting effect. The images showed differences in morphology of the imprinted and non-imprinted polymers. The **MIP8** and **NIP8** particles obtained in the bulk polymerization have irregular shapes and sizes, with a diameter of approximately 10 μm or more. The surface of **MIP8** is more porous with a lot of small cavities. The surface of **NIP8** is more uniform and smooth with only a small number of small cavities (Figure 4).

Next, we observed the surface of imprinted particles which had the highest and the lowest binding capacity of homoveratric acid. The highest capacity was noted for the copolymer of *N*-allylpiperazine and EGDMA in DMF (**MIP5**), and the lowest capacity was noted for the copolymer of allylamine and TGDMA in DMF (**MIP8_a_**).

**Figure 5:**
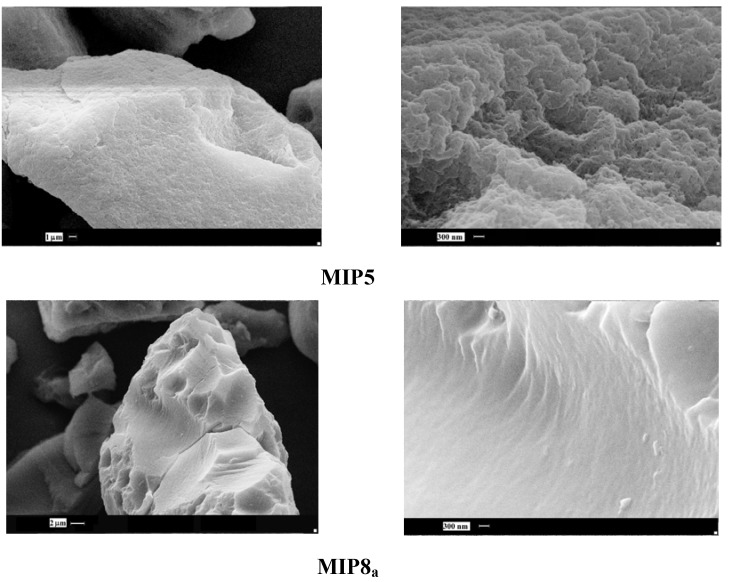
SEM micrographs of MIP**5** and MIP**8_a_** particles.

As it could be seen (Figure 5), the observable differences are significant. The surface of **MIP5** is rough, with a number of holes and cavities, but the surface of **MIP8_a_** is very smooth, with no visible cavities at all. The drastic difference in the morphology explains the different binding capacity of the particles.

### 2.6. IR Spectra of Selected Polymers

FTIR spectroscopy was applied to detect the monomers in the polymer matrix. We have selected two pair of polymers **MIP1/NIP1** and **MIP8/NIP8** which were prepared from the monomers showing the absorption bands different from those of the cross-linker. These absorption bands should derive from pyridine ring in polymers **MIP1/NIP1**, and from the amine group in polymers **MIP8/NIP8**. In the IR spectra of **MIP1/NIP1** the pyridine ring is represented by weak bands located between 3020–3080 cm^−1^ (ν_C-H_), and quite strong bands located at 1450, 1490 and 1591 cm^−1^ (ν_C=C_ and ν_C=N_). In the IR spectra of **MIP8/NIP8** the alkylamine grouping is represented by two bands 3406 and 3564 cm^−1^ (ν_sym_ and ν_asym_ N-H), 1635 cm^−1^ (δ_NH2_) and 1084 cm^−1^ (ν_C-N_). We have not observed distinct differences in the location of the absorption bands in the IR spectra of MIPs and NIPs. The IR spectra has allowed us to detect the monomers in the selected polymer matrix.

### 2.7. Molecular Recognition Mechanism

**Figure 6:**
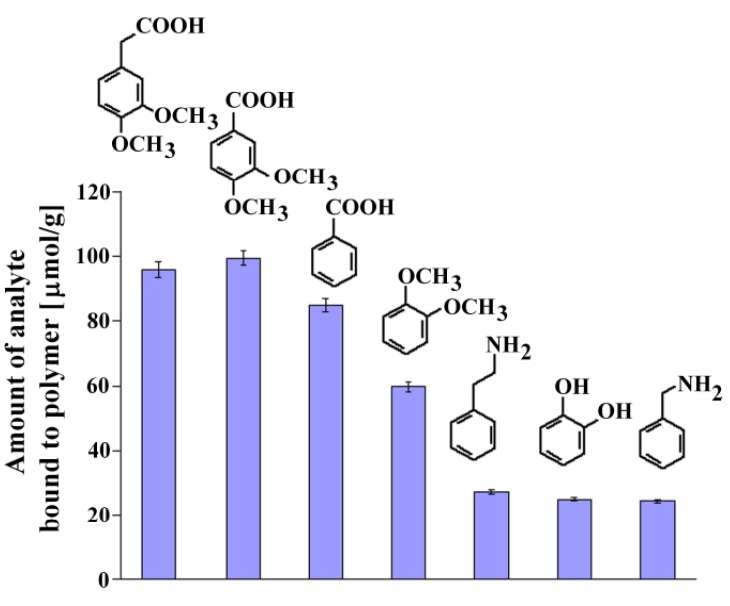
Chemical formulas and amounts of each compound bound to **MIP8** in the non-competitive binding experiments.

Recognition studies were performed using the set of structurally related compounds in non-competitive dynamic binding experiments (see Experimental). The chemical formulas of the analyzed compounds are shown in Figure 6, together with the columns presenting the amount of the corresponding analyte bound.

The affinity of homoveratric acid (95.88 ± 2.31 μmol/g) towards **MIP8** was very similar to the affinity of 3,4-dimethoxybenzoic acid (99.40 ± 2.40 μmol/g). The lowest affinities were observed for 2-phenylethylamine, 1,2-dihydroxybenzene, and benzylamine (within the range 27.12 ± 0.65–24.40 ± 0.59 μmol/g).

This observation suggests that the strong interactions between the amino groups of the polymer matrix and the carboxylic acid group in the analyte are responsible for the recognition process. Higher affinity of veratrole than 1,2-dihydroxybenzene could be the proof that methoxy groups also take a part in the molecular recognition mechanism.

### 2.8. Selectivity Toward Dopamine and Its Metabolites

**Figure 7:**
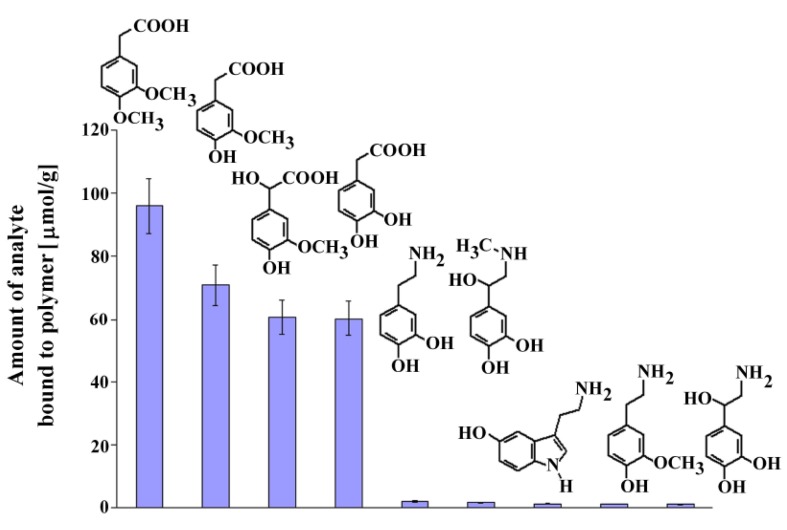
Chemical formulas and amounts of each compound bound to **MIP8** in the non-competitive binding experiments.

Next, we performed experiments similar to those described above to estimate the selectivity of **MIP8** toward dopamine, its metabolites and other compounds that could exist in the biological samples: serotonin, epinephrine, norepinephrine, DOPAC, homovanillic acid, 3-methoxytyramine, and vanilmandelic acid. The chemical formulas of the analyzed compounds are shown in Figure 7 together with the columns presenting the amount bound of the corresponding analyte.

The results demonstrated that homoveratric acid has the highest affinity to **MIP8** among all the biogenic compounds analyzed. Homovanillic acid, vanilmandelic acid, and DOPAC have significantly lower affinity, and all analyzed biogenic amines have practically no affinity to **MIP8**. The results confirmed that the prepared imprinted polymer could be used as the selective separation material, for instance in the solid phase extraction of homoveratric acid.

### 2.9. Application of MIP8 to Separation of Homoveratric Acid

We have applied 25 mg of **MIP8** as a SPE stationary phase to confirm its application for separation of homoveratric acid. The experimental details are presented in Table 3. The results showed that **MIP8** could be successfully applied as a stationary phase for SPE separation of homoveratric acid. We worked out the procedure of elution which allowed us to recover practically the total amount of homoveratric acid (in respect of the adsorbed amount) from **MIP8**. The imprinted polymer showed good enrichment properties. **MIP8** bound homoveratric acid from 20 mL of loading solution, and allowed us to produce a concentrated homoveratric acid eluate of 4 mL

**Table 3:** Separation of homoveratric acid with **MIP8** as the stationary phase in solid phase extraction.

Extraction Steps	MIP8
	Found [nmol]
1. Conditioning (2 mL, water)	< L.Q.^a^
	Bound [nmol]
2. Loading (total of 10 × 2 mL, 75 μmol/L)	829 ± 32
	Found [nmol]
3. Washing (1 mL, water)	69.5 ± 2.7
4. Eluting (total of 2 × 2 mL, methanol)	832 ± 32

^a^ below limit of quantification.

#### 2.9.1. Preliminary Analysis of Homoveratric Acid Separation from Artificial Urine

Finally, the influence of sample composition on the binding properties of **MIP8** was examined using two artificial urine formulas **AU1** [30] and **AU2** [31], both spiked with homoveratric acid. Urine is known to be a complex matrix generally containing different inorganic salts, urea, creatinine, macromolecular compounds and the typical analytical procedure requires ultrafiltration and dilution. Therefore, for many analytical applications artificial urine is widely used for *in vitro* experiments [32]. Both selected AU formulas have different compositions: **AU1** contains urea, creatinine and inorganic salts, but **AU2** contains various inorganic salts at concentrations above the physiological limits, but does not contain urea or creatinine. We performed the solid phase extraction procedure as described before (see Experimental). The obtained results are presented in Table 4.

**Table 4:** Separation of homoveratric acid using **MIP8** in the SPE procedure from spiked artificial urines **AU1** and **AU2**.

Steps	AU1	AU2
	Found [nmol]	
1. Conditioning (2 mL, water)	< L.Q.^a^	< L.Q.^a^
	Bound [nmol]	
2. Loading (total of 10 × 2 mL, 75 μmol/L)	168.0 ± 6.4	317 ± 12
	Found [nmol]	
3. Washing (1 mL, water)	28.3 ± 1.1	36.0 ± 1.4
4. Eluting (total of 2 × 2 mL, methanol)	157 ± 6.0	312 ± 12

^a^ below limit of quantification.

The results show that binding capacity of homoveratric acid to **MIP8** decreased in both artificial urines, but it was still sufficient for its separation. Marked interference with the adsorption due to urea and/or creatinine was found. Any similar effect of inorganic salts was less apparent. The recovery of homoveratric acid from the column was high, and the feasibility of carrying out an assay based on **MIP8** was proven. 

## 3. Experimental

### 3.1. General

3,4-Dimethoxyphenylacetic acid (homoveratric acid), 3,4-dimethoxybenzoic acid, 3,4-dihydroxy-phenylacetic acid, 4-hydroxy-3-methoxyphenylacetic acid (homovanillic acid), and D/L-*α*,4-dihydroxy-3-methoxyphenylacetic acid (D/L-vanilmandelic acid) were purchased from Alfa Aesar (Karlsruhe, Germany), 1,2-dimethoxybenzene, and 1,2-dihydroxybenzene were from Aldrich (Steinheim, Germany), benzoic acid, and 2-phenylethylamine were from POCh (Gliwice, Poland), 2-(4-hydroxy-3-methoxyphenyl)ethylamine (3-methoxytyramine), L-3,4-dihydroxy-α-(methylamino-methyl)benzyl alcohol D-hydrogen bitartrate salt (L-epinephrine), 3-(2-aminoethyl)-5-hydroxyindole hydrochloride (serotonin) were from Sigma (Steinheim, Germany), 2-(3,4-dihydroxyphenyl)ethylamine hydrochloride (dopamine), (±)-1-(3,4-dihydroxyphenyl)-2-aminoethanol hydrochloride (D/L-nor-epinephrine), and benzylamine were from Fluka (Steinheim, Germany). The functional monomers 2-vinylpyridine (**1**), 4-vinylpyridine (**2**), 1-vinylimidazole (**3**), allylamine (**8**) were from Fluka (Steinheim, Germany), allylurea (**6**) and allylthiourea (**7**) were from Aldrich (Steinheim, Germany), *N*-allylpiperazine (**5**) was from Alfa Aesar (Karlsruhe, Germany), and *N*-allylaniline (**4**) was supplied by Sigma (Steinheim, Germany). The cross-linkers ethylene glycol dimethacrylate (EGDMA) and triethylene glycol dimethacrylate (TGDMA) were from Fluka (Steinheim, Germany), trimethylolpropane trimethacrylate (TRIM) was from Aldrich (Steinheim, Germany). The porogen and solvents *N*,*N*-dimethylformamide (DMF), methanol, and acetone were from POCh (Gliwice, Poland). The polymerization reaction initiator 1,1’-azobiscyclohexanecarbonitrile (CHC) was from Fluka (Steinheim, Germany). Perchloric acid (60%) was from Merck (Darmstadt, Germany). The monomers were purified (if necessary) prior to use by standard procedures (vacuum distillation or recrystallization from an appropriate solvent). All other reagents were used without further purification. Ultra-pure water was delivered from a Milli-Q purification system (Millipore, France) and was used to prepare all the water solutions.

The stock solutions of the analyzed compounds were prepared by accurately weighting the appropriate amount of each compound and dissolving it in methanol or water adjusted to pH 3 with 0.04 M perchloric acid (dopamine, 3-methoxytyramine, D/L-norepinephrine, L-epinephrine, and serotonin) to give a final concentration of 10 mmol/L. Exceptions were benzoic acid, for which 25 mmol/L concentration solution was prepared and benzylamine and 2-phenylethylamine, for which concentrations of 100 mmol/L were prepared.

The standard solutions were prepared prior to use by dilution of the appropriate stock solutions with ultra-pure water to obtain the desired concentrations. All stock solutions were stored in the dark at +8 °C. 

The UV-Vis measurements were performed with a UV-1605PC spectrophotometer (Shimadzu, Germany). The calibration lines as a function of absorbance (y) versus concentration (x) were constructed at λ_max__._ of the investigated compounds. Each point was measured in triplicate. The linearity of calibration lines was good (r^2^ > 0.997). The wavelengths (λ_max_ in nm), the limits of quantification (LOQ in μmol/L), and the limits of detection (LOD in μmol/L) were as follows: homoveratric acid (278, 7.39, 2.44), benzoic acid (273, 23.31, 7.69), 3,4-dimethoxybenzoic acid (290, 5.89, 1.94), 1,2-dihydroxybenzene (275, 8.71, 2.88), 1,2-dimethoxybenzene (273, 9.23, 3.05), 3,4-dihydroxy-phenylacetic acid (280, 3.86, 1.27), homovanillic acid (279, 12.50, 4.13), (D/L) vanilmandellic acid (279, 10.36, 3.42), benzylamine (257, 178.32, 58.85), 2-phenylethylamine (258, 217.25, 71.69), 3-methoxytyramine (279, 5.82, 1.92), dopamine (280, 6.26, 2.07), D/L-norepinephrine (279, 4.62, 1.52), L-epinephrine (279, 5.28, 1.74), and serotonin (276, 7.67, 2.53). IR spectra were recorded in KBr pellets on a FT IR Perkin Elmer SPECTRUM 1000 instrument. The scanning electron micrographs were taken at the Department of Chemistry, University of Warsaw, Poland. The surface of imprinted and non-imprinted materials were studied on a LEO 435VP microscope (Zeiss, Germany). All samples were Au/Pd sputtered-coated before analysis.

### 3.2. Molecular Modeling

The following compounds were used in the studies: 2-vinylpyridine (**1**), 4-vinylpyridine (**2**), 1-vinylimidazole (**3**), *N*-allylaniline (**4**), *N*-allylpiperazine (**5**), allylurea (**6**), allylthiourea (**7**), allylamine (**8**) as the monomers, and homoveratric acid as the template. Three-dimensional structures were drawn using the Hyperchem version 7.01 software [33]. The starting structure of homoveratric acid was created on the basis of the published crystallographic data [34,35]. Geometries of all structures were optimized using the semiempirical PM3 method until the energy gradient was below 0.01 kcal/mol Å. The theoretical prepolymerization complex systems were built up from four monomer molecules and one molecule of homoveratric acid taking into account the molar ratio used in the synthetic procedure. The complexes were constructed manually by placing the monomer molecules in the proximity of functional groups of the template, in such a way that the formation of as many hydrogen bonding interactions as possible was allowed between the monomers and template functional groups. Starting distances between the atoms involved in the interactions were 2.5–3.0 Å. The enthalpies of formation obtained for optimized structures were used in discussion (Δ*H_complex_*), and to calculate the energies of complexation reaction (Δ*E*)—see the equation (3) in part 2.1.2.

### 3.3. Preparation of Homoveratric Acid Imprinted Polymer

The experimental amounts of reagents (moles, masses and volumes) used for the preparation of the different types of polymers are listed in Table 4. The MIPs coded as **MIP1**–**MIP8**, **MIP8_a,b_** were prepared by the radical bulk polymerization. Briefly, homoveratric acid as the template, the selected functional monomer and the cross-linker were dissolved in DMF acting as the porogen (1 mL of the porogen to 1 mL of the sum of monomer and the cross-linker) in thick-walled glass tubes. The molar ratio of the template to the functional monomer and the cross-linker was equal to 1:4:20 for EGDMA, TGDMA and 1:4:4 for TRIM. Next, the initiator (CHC) was added. The homogeneous solutions were purged with nitrogen for ca. 3–5 min and then glass tubes were sealed. Subsequently, the polymerization was carried out under a nitrogen atmosphere for 24 h at 88 °C. Yields of the crude polymers were almost 100 %. The substrates were not detected by UV spectroscopy in the solvent after template extraction from polymer particles*.*

**Table 4:** Selected details of the polymerization processes.

**No of MIPs**	Template mg [mmol]	Functional Monomermg [mmol]	Cross-linkermL [mmol]	Initiatormg	PorogenmL
**1**		2-vinylpyridine (**1**)	EGDMA	CHC	DMF
	39.2 [0.2]	84.1 [0.8]	0.754 [4.0]	14.1	0.841
**2**		4-vinylpyridine (**2**)	EGDMA	CHC	DMF
	39.2 [0.2]	84.1 [0.8]	0.754 [4.0]	14.1	0.841
**3**		1-vinylimidazole (**3**)	EGDMA	CHC	DMF
	39.2 [0.2]	75.3 [0.8]	0.754 [4.0]	14.1	0.827
**4**		*N*-allylaniline (**4**)	EGDMA	CHC	DMF
	39.2 [0.2]	106.6 [0.8]	0.754 [4.0]	14.1	0.863
**5**		*N*-allylpiperazine (**5**)	EGDMA	CHC	DMF
	39.2 [0.2]	101.0 [0.8]	0.754 [4.0]	14.1	0.866
**6**		allylurea (**6**)	EGDMA	CHC	DMF
	39.2 [0.2]	80.1 [0.8]	0.754 [4.0]	14.1	0.754
**7**		allylthiourea (**7**)	EGDMA	CHC	DMF
	39.2 [0.2]	92.9 [0.8]	0.754 [4.0]	14.1	0.754
**8**		allylamine (**8**)	EGDMA	CHC	DMF
	39.2 [0.2]	45.7 [0.8]	0.754 [4.0]	14.1	0.814
**8_a_**			TGDMA	CHC	DMF
		allylamine (**8**)	1.145 [4.0]	14.1	1.109
**8_b_**	39.2 [0.2]	45.7 [0.8]	TRIM	CHC	DMF
			0.271 [0.8]	4.7	0.331

The bulk rigid polymers were ground in a mortar with a pestle and wet-sieved into particles below 45 μm diameter. Fine particles were separated by repeated decantation in acetone. Homoveratric acid molecules were then removed from the polymer particles using the continuous extraction process in a Soxhlet apparatus (24–36 h, 80 mL, methanol/water 85/15 ^v^/_v_) and dried under vacuum at room temperature. Homoveratric acid removal was controlled by UV-Vis spectroscopy. Non-imprinted polymers (**NIP1**–**NIP8**, **NIP8_a,b_**) were prepared under the same polymerization conditions but without the template molecule and were treated in the same way as the corresponding imprinted polymers.

### 3.4. Binding Experiments

The stationary binding experiments were performed to evaluate the binding ability of MIPs and NIPs particles. Polypropylene tubes of 10 mL were filled with 10 mg of **MIP1**–**MIP8**, **MIP8_a,b_** or **NIP1**–**NIP8**, **NIP8_a,b_**. To each tube aqueous homoveratric acid standard solutions of 24 μmol/L concentration (5 mL) was added. The tubes were sealed and oscillated by a shaker (Heidolph, Germany) at room temperature for 24 h. Then, the tubes were centrifuged for 10 min at 3,000 rpm, and aliquots of supernatant (0.7 mL) were used to analyze the unbound amount of homoveratric acid by UV-Vis spectroscopy. The amount of homoveratric acid bound to the polymer was calculated by subtracting the unbound amount from the initial amount of homoveratric acid. For Scatchard analysis, polypropylene tubes were filled with 10 mg of **MIP8** or **NIP8**. Next, the different aqueous homoveratric acid standard solutions (5 mL, 0.019, 0.024, 0.03, 0.06, 0.12, 0.24, 0.6, and 1.2 mmol/L) were added to each tube. Then the polymers were treated in the same way as described above. 

The dynamic binding experiments were performed to evaluate the binding ability of selected MIPs and NIPs particles. Polypropylene SPE columns of 1 mL were filled with 25 mg of **MIP1–MIP8** or **NIP1**–**NIP8** particles and secured by glass-fiber frits. The particles were conditioned with water (2 mL) and then they were loaded (until further adsorption on the polymer particles was not observed) with successive portions (1 mL each) of 0.35 mmol/L standard solution of homoveratric acid in water (additionally, for **MIP8** and **NIP8**, 24 μmol/L solution was analyzed). The aliquots of supernatant (0.1, 0.4 or 0.7 mL) were used to analyze the unbound amount of homoveratric acid by UV-Vis spectroscopy. The bound amount of homoveratric acid was calculated by subtracting the unbound amount from the initial amount of homoveratric acid. 

The non-competitive binding experiments were carried out for the most promising polymer **MIP8** to determine its selectivity and the intermolecular interactions in the polymer matrix. Polypropylene SPE columns of 1 mL capacity were filled with 25 mg of **MIP8** particles and secured by glass-fiber frits. Then the particles were treated according to the dynamic procedure as described above. The standard solutions of the analytes (concentration 0.35 mmol/L), *viz.* benzoic acid, 3,4-dimethoxy-benzoic acid, veratrole, benzylamine, 2-phenylethylamine, 1,2-dihydroxybenzene, DOPAC, homovanillic acid, (D/L)-vanilmandelic acid, 3-methoxytyramine, dopamine, D/L-norepinephrine, L-epinephrine, and serotonin were used in the loading step. All experiments were performed in triplicate.

Solid phase extraction experiments were carried out on Macheney-Nagel SPE manifold. Polypropylene 1 mL SPE columns (Chromabond, Germany) secured by glass-fiber frits were filled with 25 mg of **MIP8**. The following SPE protocol steps were applied on each column: conditioning (water, 2 mL), loading (ten portions of 2 mL of aqueous standard solution of homoveratric acid of 75 μmol/L), washing (water, 1 mL), eluting (methanol, two portions of 2 mL). The flow rate of each SPE step was 1 mL/min. The conditioning, loading, and washing fractions were treated according to the dynamic binding procedure described above. The elution fractions were collected and aliquots of 0.7 mL were used to analyze the amount of homoveratric acid eluted from **MIP8** cartridges by UV-Vis spectroscopy. Triplicate cartridges of **MIP8** were used for each extraction.

Two artificial urines, **AU1** [30] and **AU2** [31], were prepared according to established formulas with minor modifications. The composition of **AU1** was: urea, 416 μmol/mL, creatinine, 17.70 μmol/mL, NaCl, 154 μmol/mL, NH_4_Cl, 48 μmol/mL, Na_2_SO_4_, 21.10 μmol/mL, KH_2_PO_4_, 17.60 μmol/mL, and the composition of **AU2** was: NaCl, 105.50 μmol/mL, KCl, 63.70 μmol/mL, NH_4_Cl, 36.30 μmol/mL, MgSO_4_, 3.85 μmol/mL, Na_2_SO_4_, 16.95 μmol/mL, KH_2_PO_4_, 32.20 μmol/mL. Aliquots of each artificial urine (0.2 mL) and of stock solution of homoveratric acid (75 μL) were transferred to a 10.0 mL volumetric flask and diluted to volume with ultra-pure water. Solid phase extractions experiments were carried out according to procedure described above.

## 4. Conclusions

The presented molecular imprinting procedure allowed us to obtain a homoveratric acid-imprinted polymer which exhibits highly selective binding towards homoveratric acid. The hydrogen-bonding interactions between carboxylic acid and methoxy groups in the template and amino groups in the binding sites of polymer are responsible for a molecular recognition process. Theoretical computations operformed on the prepolymerization complexes allowed a deeper analysis of the experimental results. The polymer which was prepared from allylamine as the functional monomer, ethylene glycol dimethacrylate as the cross linker, and *N*,*N*-dimethylformamide as the porogen shows the highest imprinting factor of 3.43. Scatchard analysis revealed the presence of both specific and non-specific adsorption sites in the polymer matrix. The thus obtained polymer could be useful for the separation of homoveratric acid in SPE mode. 
